# Differences in the management of patients requiring an emergency resection for colonic cancer in two European populations

**DOI:** 10.1093/bjsopen/zrac126

**Published:** 2022-10-19

**Authors:** John C Taylor, Lene H Iversen, Dermot Burke, Paul J Finan, Mark M Iles, Eva J A Morris, Philip Quirke, YCR BCIP Study Group, K Absolom, D Swinson, D Tolan, S Howell, S Alderson, A Glover, A Hindley, H Rossington, J Mara, E Boldison

**Affiliations:** Leeds Institute of Medical Research at St James’s, University of Leeds, Leeds, UK; Leeds Institute for Data Analytics, University of Leeds, Leeds, UK; Department of Surgery, Aarhus University Hospital, and Danish Colorectal Cancer Group, Aarhus, Denmark; Leeds Institute of Medical Research at St James’s, University of Leeds, Leeds, UK; Leeds Institute of Medical Research at St James’s, University of Leeds, Leeds, UK; Leeds Institute for Data Analytics, University of Leeds, Leeds, UK; Leeds Institute of Medical Research at St James’s, University of Leeds, Leeds, UK; Leeds Institute for Data Analytics, University of Leeds, Leeds, UK; Nuffield Department of Population Health, Big Data Institute, University of Oxford, Oxford, UK; Leeds Institute of Medical Research at St James’s, University of Leeds, Leeds, UK

## Abstract

**Background:**

Patients with colonic cancer who require emergency colonic cancer surgery often experience poorer outcomes compared with their elective counterparts. In this setting, several treatments approaches are available. In 2009, Danish guidelines recommended treatment with stent for obstruction in left-sided tumours as a bridge to surgery, if expertise is accessible. The aim of this study was to compare the use of elective and emergency resections for colonic cancer and postoperative mortality in two similar demographic populations.

**Methods:**

All patients who underwent a major resection for colonic cancer, between 2005 and 2016 in Denmark and Yorkshire (UK) were identified. The proportion undergoing emergency surgery, the proportion receiving a stent procedure before their resection, and 30-day postoperative mortality were compared between the populations. Logistic regression was used to determine changes in the proportion of those undergoing emergency surgery and 30-day postoperative mortality.

**Results:**

Out of 45 397 patients treated during the study interval, 41 880 were selected. Emergency surgery decreased in Denmark from 16.6 per cent in 2005–07 to 12.9 per cent in 2014–16, but increased in Yorkshire (13.5 per cent to 16.8 per cent). Danish patients with left-sided tumours were less likely to undergo emergency surgery (risk ratio 0.90, 95 per cent c.i. 0.82 to 0.99) and an increase in stent use coincided with a statistically significant decrease in emergency surgery in these patients. Thirty-day postoperative mortality in all resections (elective and emergency) decreased in both populations, but a larger decrease was observed in Denmark (7.7 per cent to 3.0 per cent in Denmark and 7.1 per cent to 3.3 per cent in Yorkshire).

**Conclusion:**

Patients in Denmark experienced a reduction in the use of emergency resection and increase in stenting procedures, following the policy implemented in some departments of converting potential emergency resections into elective resections.

## Teaser

This study is part of a large educational intervention programme with an ambitious aim of improving outcomes for colorectal patients in the Yorkshire region of UK (the Yorkshire Cancer Research Bowel Cancer Improvement Programme). The focus of this particular paper compares the use of emergency surgery in patients with colonic cancer from the region with their counterparts in Denmark, and how this may influence the amount of emergency surgery taking place. Patients in Denmark, but not Yorkshire, experienced a reduction in the use of emergency resection, which coincided with an increase in stenting procedures.

## Introduction

Approximately 15–30 per cent of patients with colorectal cancer present as an emergency^1^. Those who require an emergency surgical resection tend to have worse short-term outcomes than those who receive elective care, and these include increased rates of postoperative mortality, postoperative complications, and length of hospital stay^2–5^. Poorer survival and recurrence rates have also been reported in some, but not all studies^6–8^. The introduction of alternative treatments for suitable patients, such as, self-expanding metallic stents (SEMs) as a bridge to surgery, may reduce the number of patients undergoing an emergency resection^9^; however, because of the expertise required and the lack of consensus in published guidelines, the utilization of treatment options such SEMs are likely to vary both within and across populations^10,11^.

Comparisons in the management of patients with colorectal cancer between similar demographic populations, can help to identify differences in practices, which may have an impact on outcomes^12,13^, but few studies have been undertaken. The Yorkshire Cancer Research Bowel Cancer Improvement Programme (YCR BCIP) is using this philosophy by attempting to identify areas of improvement in the management of patients with colorectal cancer in Yorkshire^14^. Denmark and the UK both have healthcare provision that is largely free at the point of care and financed through taxation, with a similar disease burden and adult life expectancy^15,16^. This, and given that Denmark has a similar population size to Yorkshire (both 5.7 million), presents the opportunity to use Denmark as a suitable comparator in the management of patients with colorectal cancer.

In 2009, guidelines were issued in Denmark recommending treatment with SEMs for obstruction in left-sided tumours as a bridge to surgery^17^. Given the demanding technical skill, however, it was emphasized that the use of stents in an emergency situation should only be performed in departments with the necessary expertise.

The present study was undertaken to compare the use of elective and emergency resections for colonic cancer and postoperative mortality between Denmark and the region of Yorkshire, UK.

## Methods

This was a retrospective population-based study of patients with a first primary colonic cancer diagnosed in Denmark and the region of Yorkshire, UK. All patients aged 18 years or older were diagnosed between 1 January 2005 and 31 December 2016 (ICD-10 C18 and C19) and had undergone a major surgical resection at a Danish or an English NHS hospital, up to 1 year after diagnosis. Patients with a malignant neoplasm of the appendix (ICD-10 C18.1) were excluded as these were not recorded in Denmark from 2014.

Danish patients were obtained from the Danish Colorectal Cancer Group (DCCG) database^18^, which captures all patients with colorectal cancer who have been diagnosed and/or treated at a public hospital. The main surgical procedure, and urgency of that procedure, is recorded in the DCCG and those patients recorded as undergoing a major resection procedure were identified and categorized as either an elective or emergency resection. Major resection was defined as receiving selected radical procedures (*Table S1*) occurring within 1 month before, and up to 1 year after, the date of diagnosis. No formal definition of emergency surgery is given in the DCCG, except that the indication for emergency surgery (ileus, perforation, bleeding, or otherwise) should be reported. The majority of those classed as an emergency are operated on within 36 h of admission. Surgical procedures, and other interventions, before the main surgery are also recorded and those who had a stent inserted before their main surgical procedure were identified.

Patients in Yorkshire were identified using the UK Colorectal Cancer Intelligence Hub’s COloRECTal Repository (CORECT-R) by linkage of the data from the national cancer registry (National Cancer Registration and Analysis Service) and inpatient hospital admissions (Hospital Episode Statistics, HES)^19^. Major surgical resection is defined in CORECT-R using a methodology that matches procedure codes within HES to identify all operations used to surgically treat colorectal cancer. The nature of the patients’ admission for the procedure is also recorded. A resection is categorized as an emergency resection if the procedure was undertaken within 2 days of an emergency admission, and an elective resection otherwise. Those who had a recorded HES procedure with OPSC4 codes pertaining to the insertion of a stent (H214, H243, H244, H273, H274, H314, and Y141 to Y149) up to 30-days before, or with the same date as the major surgical resection, were identified as having a stent inserted.

### Outcomes of interest

The primary outcome of interest was the rate of emergency resection in the two populations over time. Secondary outcomes included the rate of stent procedures, hospital variation in the use of emergency resection, and 30-day postoperative mortality following elective and emergency resections by study interval, and defined as death of the patient within 30-days of the resection date.

### Statistics

The proportion of emergency resections was calculated as a percentage of all resections over the study interval and by grouped year of diagnosis (2005–2007, 2008–2010, 2011–2013, and 2014–2016). To investigate the factors associated with use of emergency resection and to test the statistical significance of changes over time, Danish and Yorkshire populations were modelled separately using multilevel mixed effects. Poisson regression with a robust error variance to estimate the risk ratio (RR), as is recommended for binary outcomes where the probability of the outcome is common^20^. The binary dependent variable was emergency resection or not, independent fixed effects were age group (18–59, 60–69, 70–79, and 80 years or older), sex, tumour site (right, left, or unspecified), stage of disease (1–4) and study interval, and hospital where the operation was conducted was fitted as a random effect. Tumours located in the caecum, ascending colon, hepatic flexure, and transverse colon were categorized as right-sided tumours, whereas those in the splenic flexure, descending colon, sigmoid colon, and rectosigmoid junction were categorized as left-sided tumours. Stage of disease was missing in 2.4 per cent and 4.0 per cent of Danish and Yorkshire patients respectively. Therefore, an ordered logistic regression was used to impute missing values and estimated model coefficients and standard errors according to Rubin’s combination rules.

Variation in the use of emergency resection by hospital was assessed by the median OR (MOR), calculated from the estimated variance of the distribution of random effects after fitting of multilevel logistic models with the same covariates described above and stratified by study interval. The MOR quantifies the variation areas between the second-level variation (hospitals in the models) and allows comparisons with the fixed effects covariates on the OR scale^21,22^. A MOR equal to one would indicate no variation in the use of emergency surgery between hospitals, whereas a MOR more than one would indicate variation. Bootstrapping was performed to calculate biased-corrected 95 per cent confidence intervals for the variance estimate from the multilevel logistic models using 200 replications, which were then used to create confidence intervals for the MORs^23^.

To moderate the effect of colorectal screening programmes on changes in the emergency resection proportion, for all analyses, Danish patients who were registered as being diagnosed based on the Danish Screening Programme and Yorkshire patients who had a screening diagnosis as defined by the Routes to Diagnosis methodology^24^ were excluded.

The RR for deaths following emergency resection were calculated and compared with elective resection by fitting a multilevel mixed-effects regression model, with hospital as a random effect. The binary dependent variable was 30-day postoperative mortality, and independent fixed effects were age, sex, tumour site, stage of disease, and surgical urgency (elective or emergency resection), while stratifying by study interval. Adjustments were performed for these covariates, as they have been previously associated with both postoperative mortality^25^ and use of emergency surgery^3^ so are assumed to be confounders of the relationship between them. The missing stage information were imputed as described above.

Statistical analyses were performed using STATA version 16, (StataCorp, College Station, TX, USA). Statistical significance was set at *P* < 0.05.

## Results

### Emergency resections

Out of 45 397 patients treated, a total of 24 828 and 17 052 major resections were included for patients diagnosed with colonic cancer between 2005 and 2016 in Denmark and Yorkshire respectively (*Table 1*). Exclusions consisted of 1742 patients in Denmark (1733 elective and 9 emergency) and 1775 patients in Yorkshire (1752 elective and 23 emergency) with a diagnosis derived from the respective screening programmes.

**Table 1:** Characteristics for patients with colonic cancer undergoing an elective and emergency resection in Denmark and Yorkshire between 2005 and 2016

		Denmark				Yorkshire			
		Elective		Emergency		Elective		Emergency	
		*n*	%	*n*	%	*n*	%	*n*	%
Total		21 053	100	3775	100	14 500	100	2552	100
Age (years)	18–59	3131	14.9	523	13.9	2492	17.2	495	19.4
	60–69	5717	27.2	909	24.1	3336	23.0	587	23.0
	70–79	7464	35.5	1166	30.9	5165	35.6	786	30.8
	≥80	4741	22.5	1177	31.2	3507	24.2	684	26.8
Sex ratio	M:F	10 422:10 631	49.5:50.5	1757:2018	46.5:53.5	7757:6743	53.5:46.5	1389:1163	54.4:45.6
Tumour site	Right	10 460	49.7	2076	55.0	7118	49.1	1208	47.3
	Left	10 584	50.3	1695	44.9	7024	48.4	1258	49.3
	Unspecified	<10	<1	<10	<1	358	2.5	86	3.4
Stage	1	3060	14.5	76	2.4	1898	13.1	58	2.3
	2	8485	40.3	1169	31.0	5614	38.7	887	34.8
	3	6273	29.8	1173	31.1	5004	34.5	1022	40.0
	4	2820	13.4	1228	32.5	1363	9.4	523	20.5
	Unknown	415	2.0	129	3.4	621	4.3	62	2.4
Study interval	2005–2007	5046	24.0	1003	26.6	3892	26.8	607	23.8
	2008–2010	5029	23.9	955	25.3	3808	26.3	626	24.5
	2011–2013	5414	25.7	993	26.3	3523	24.3	656	25.7
	2014–2016	5564	26.4	824	21.8	3277	22.6	663	26.0

Emergency resections accounted for 15.2 per cent of all resections in Denmark and 15.0 per cent in Yorkshire.

The use of emergency resections increased with increasing age and stage of disease in both Denmark and Yorkshire (*Table 2*). Patients with a left-sided tumour were significantly less likely to have received an emergency resection than those with a right-sided tumour in Denmark (adjusted RR 0.90, 95 per cent c.i. 0.82 to 0.99, *P* = 0.024) but not in Yorkshire (RR 1.11, 95 per cent c.i. 0.99 to 1.24, *P* = 0.062). Use of emergency resections decreased over time in Denmark from 16.6 per cent in 2005–2007 to 12.9 per cent in 2014–2016, but significantly increased in Yorkshire from 13.5 per cent to 16.8 per cent (*P* < 0.001).

**Table 2:** Adjusted risk ratios and 95 per cent confidence intervals for use of emergency resection in patients with colonic cancer in Denmark and Yorkshire between 2005 and 2016

		Denmark			Yorkshire		
		Emergency (%)	RR (95% c.i.)	*P*	Emergency (%)	RR (95% c.i.)	*P*
Total		15.2	–	–	15.0	–	–
Age (years)	18–59	14.3	0.99 (0.90, 1.10)	0.872	16.6	1.06 (0.93, 1.20)	0.393
	60–69	13.7	1.00 (reference)	–	15.0	1.00 (reference)	–
	70–79	13.5	1.02 (0.92, 1.11)	0.805	13.2	0.94 (0.83, 1.05)	0.248
	≥80	19.9	1.49 (1.36, 1.64)	<0.001	16.3	1.16 (1.03, 1.31)	0.014
Sex	Male	14.4	1.00 (reference)	–	15.2	1.00 (reference)	–
	Female	16.0	1.05 (0.96, 1.14)	0.276	14.7	0.97 (0.92, 1.02)	0.240
Tumour site	Right	16.6	1.00 (reference)	–	14.5	1.00 (reference)	–
	Left	13.8	0.90 (0.82, 0.99)	0.024	15.2	1.11 (0.99, 1.24)	0.062
	Unspecified	NA	NA	–	19.4	1.39 (1.09, 1.77)	0.007
Stage	1	2.4	0.22 (0.18, 0.28)	<0.001	3.0	0.23 (0.18, 0.29)	<0.001
	2	12.1	1.00 (reference)	–	13.6	1.00 (reference)	–
	3	15.8	1.34 (1.28, 1.40)	<0.001	17.0	1.25 (1.15, 1.37)	<0.001
	4	30.3	2.58 (2.35, 2.83)	<0.001	27.7	2.02 (1.84, 2.22)	<0.001
Study interval	2005–2007	16.6	1.00 (reference)	–	13.5	1.00 (reference)	–
	2008–2010	16.0	0.98 (0.89, 1.08)	0.674	14.1	1.04 (0.92, 1.18)	0.540
	2011–2013	15.5	0.99 (0.87, 1.13)	0.926	15.7	1.16 (1.00, 1.34)	0.051
	2014–2016	12.9	0.86 (0.71, 1.03)	0.106	16.8	1.24 (1.10, 1.39)	<0.001

NA, not available; RR, risk ratio.

### Tumour site and use of stents

**Fig. 1:**
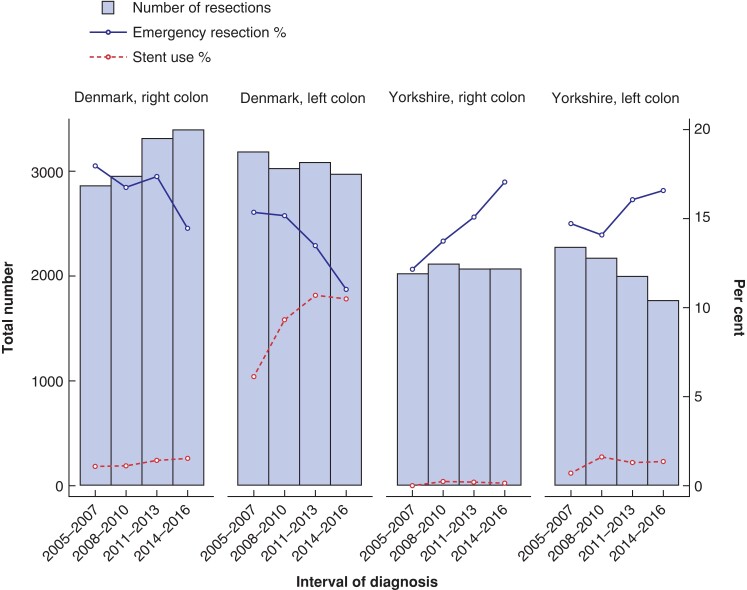
Number of resections, proportion of resections classified as an emergency and proportion of resections that were preceded by a stent procedure for patients with left- and right-sided colonic cancer in Denmark and Yorkshire by time interval

Use of emergency resection decreased in both patients with right- and left-sided tumours in Denmark; however, the decrease was significant in the left-sided tumours (*P* = 0.520 and *P* = 0.007 respectively; *Table 3* and *Fig. 1*). In Yorkshire, no significant change in use of emergency resection was observed in patients with left-sided tumours (*P* = 0.327), but an increase was seen in those with right-sided tumours (*P* < 0.001).

**Table 3:** Adjusted risk ratios and 95 per cent confidence intervals for emergency resection in patients with right- and left-sided colonic cancer in Denmark and Yorkshire between 2005 and 2016

Population	Tumour site	Study interval	Elective	Emergency		RR (95% c.i.)	*P*
			*n*	*n*	% of all resections		
Denmark							
	Right-sided	2005–2007	2349	514	18.0	1.00 (reference)	–
		2008–2010	2460	495	16.8	0.97 (0.84, 1.11)	0.659
		2011–2013	2743	576	17.4	1.06 (0.90, 1.24)	0.481
		2014–2016	2908	491	14.4	0.93 (0.74, 1.16)	0.520
							
	Left-sided	2005–2007	2697	489	15.3	1.00 (reference)	–
		2008–2010	2569	460	15.2	0.98 (0.88, 1.11)	0.780
		2011–2013	2671	417	13.5	0.93 (0.81, 1.05)	0.237
		2014–2016	2647	329	11.1	0.77 (0.64, 0.93)	0.007
Yorkshire							
	Right-sided	2005–2007	1779	246	12.1	1.00 (reference)	–
		2008–2010	1829	291	13.7	1.12 (0.91, 1.39)	0.285
		2011–2013	1757	312	15.1	1.25 (1.02, 1.53)	0.029
		2014–2016	1718	353	17.0	1.40 (1.18, 1.66)	<0.001
							
	Left-sided	2005–2007	1942	335	14.7	1.00 (reference)	–
		2008–2010	1868	306	14.1	0.96 (0.83, 1.10)	0.518
		2011–2013	1679	321	16.1	1.08 (0.88, 1.33)	0.451
		2014–2016	1476	293	16.6	1.09 (0.91, 1.31)	0.327

RR, risk ratio.

The observed proportion of patients receiving a stent procedure increased for patients with a left-sided tumour in Denmark from 6.1 per cent in 2005–2007 to 10.5 per cent in 2014–2016, while remaining less than 2 per cent for patients with a right-sided tumour (*Fig. 1*). A small increase in the proportion of patients receiving a stent procedure was observed for left-sided Yorkshire patients (0.7 per cent to 1.4 per cent).

### Hospital variation

The MOR for the use of emergency resection across Danish hospitals increased from 1.18 (95 per cent c.i. 1.08 to 1.24) in 2005–2007 to 1.52 (95 per cent c.i. 1.42 to 1.59) in 2014–2016. There was no increase in the MOR across Yorkshire hospitals; 1.19 (95 per cent c.i. 1.06 to 1.24) in 2005–2007, and 1.24 (95 per cent c.i. 1.14 to 1.32) in 2015–2017.

### Postoperative mortality

Thirty-day postoperative mortality for all patients undergoing an elective or emergency resection decreased over time in both Denmark (7.7 per cent to 3.0 per cent) and Yorkshire (7.1 per cent to 3.3 per cent). For patients who underwent an emergency resection, a larger decrease in 30-day mortality was observed over the study interval in Yorkshire (16.2 per cent to 7.7 per cent) than that for Denmark (18.0 per cent to 11.5 per cent); however, a larger decrease in 30-day mortality was observed in Denmark (5.7 per cent to 1.8 per cent) than Yorkshire (5.7 per cent to 2.4 per cent) for patients who underwent an elective resection.

The adjusted RRs for 30-day mortality in patients who underwent an emergency resection compared with elective resection, stratified by study interval are reported in *Table 4*. In Denmark, patients undergoing an emergency resection were more likely to have died than those undergoing an elective resection in 2005–2007, RR 2.55 (95 per cent c.i. 2.09 to 3.10), and this had increased by 2014–2016 to RR 4.67 (95 per cent c.i. 3.44 to 6.33). In Yorkshire, the corresponding change was not to the same extent: RR 2.60 (95 per cent c.i. 1.96 to 3.46) in 2005–2007, to RR 3.13 (95 per cent c.i. 2.36 to 4.14) in 2014–2016.

**Table 4:** Observed 30-day mortality rates for elective and emergency resections, and risk ratios and 95 per cent confidence intervals for 30-day mortality in emergency resections compared with elective resection in patients with colonic cancer in Denmark and Yorkshire by time interval

Population	Study interval	Observed 30-day mortality (%)			Emergency *versus* elective	
		Elective	Emergency	All	RR (95% c.i.)	*P*
Denmark						
	2005–2007	5.7	18.0	7.7	2.55 (2.09, 3.10)	<0.001
	2008–2010	4.1	18.1	6.4	3.63 (2.96, 4.44)	<0.001
	2011–2013	2.7	14.5	4.5	4.30 (3.48, 5.31)	<0.001
	2014–2016	1.8	11.5	3.0	4.67 (3.44, 6.33)	<0.001
Yorkshire						
	2005–2007	5.7	16.2	7.1	2.60 (1.96, 3.46)	<0.001
	2008–2010	4.9	12.8	6.0	2.48 (1.99, 3.08)	<0.001
	2011–2013	3.2	11.7	4.6	3.36 (2.82, 4.01)	<0.001
	2014–2016	2.4	7.7	3.3	3.13 (2.36, 4.14)	<0.001

RR, risk ratio.

## Discussion

This study has identified differences in the use of emergency resection for patients with colonic cancer between Denmark and Yorkshire. Postoperative mortality in the earliest interval of the study for all patients was higher in Denmark than in Yorkshire. The substantial reduction in use of emergency resections in Denmark over the study interval coincided with a decrease in the overall 30-day mortality rate that was subsequently lower than that in Yorkshire by the latest interval of the study.

The high 30-day mortality in Danish patients undergoing emergency resection compared with those in Yorkshire, indicates that only the patients with a higher morbidity are the undergoing an emergency resection. When the models were stratified by study interval, the risk of postoperative mortality increased over time for Danish patients undergoing an emergency resection compared with those undergoing an elective resection. This provides some evidence in the shifting of Danish patients who would have undergone emergency resection to an elective resection, for those with potentially curable disease; however, further work to investigate whether this is indeed the case is required.

Differential use of SEMs as a bridge to surgery may explain the large decrease observed in the proportion of emergency resection performed in Denmark but not in Yorkshire. Danish guidelines in 2009/2010 recommended treatment with SEMs for obstruction in left-sided tumours without suspicion of perforation where possible, as a bridge to surgery^17^. Some evidence of this was observed in the present study as patients with left-sided tumours were less likely to undergo an emergency resection than those with a right-sided tumour and we observed an increase in the use of stents after 2005–2007; however, the guidelines also highlight the demanding technical skill and set-up for this technique needed in the emergency situation and that stenting should only be performed in departments with the necessary expertise. As a result, the use of SEMs increased from the early 2010s but is likely to vary by surgical department. This, and different interpretation of the existing literature may explain the wide variation of emergency surgery by treating hospital observed in the later interval of this study.

Colonic stents were also considered as a bridge to surgery in the English 2011 NICE guidelines^26^. This was updated in 2014 to state that it should be explained to patients (or family members) that the obstruction can be managed initially either by emergency surgery or colonic stent with no clear evidence that one is better than the other, and/or patients be given the chance to participate in a randomized clinical trial comparing the two treatments^27^. The observed variation in emergency surgery between Yorkshire hospitals seen in this study is also likely to reflect this. Although current evidence may indicate bridge to surgery as the preferred treatment in some cases^28–30^, the differences between the Danish and English guidelines and the timing of their releases, together with the evidence available at the time of study may explain the differences observed here. In this study, an increase in the use of stents in Yorkshire was not observed, and patients with a left-sided tumours were not less likely to receive an emergency resection. However, further investigation on the uptake of SEMs in both populations is required to determine the contribution to the amount of emergency surgery performed.

While the total number of emergency resections in Yorkshire remained relatively stable the number of elective resections decreased, resulting in a statistically significant increase in the emergency resection proportion. The English screening programme began in July 2006 but by October 2008 not all of the Yorkshire region had achieved complete roll out^31^. National coverage across all English regions was achieved in 2010. Excluding screened patients here could have impacted the number of elective cases in later intervals, as the population ‘at-risk’ of diagnosis (and hence resection) will have reduced if diagnosed earlier through screening. The Danish screening programme was introduced at a later time, in March 2014^32^. Additionally, Denmark had a higher resection rate than Yorkshire over the same study time interval^33^. This, in addition to a higher incidence rate in Denmark^34^, may also explain why the overall number of resected patients in this study was much higher in Denmark when the overall populations are of equal size.

Comparative data on patient morbidity were not available in this study. Factors such as socioeconomic deprivation, co-morbidities, ASA grade, and histopathological profile have been reported to be associated with emergency patients^1,7,35^. Data on these characteristics would have allowed us to account for their influence on the differences in the use of emergency surgery reported in this study. The potential for these factors to contribute to the differences in practice observed here, should be considered in studies that look to confirm these results.

It is important to consider the limitations of the data used here when considering the findings of this study. The main weakness of the study concerns the definition of emergency resection and how directly comparable the proportions calculated in the two populations are. Emergency resection in Denmark is recorded by surgeons using a surgical proforma to indicate the urgency of the procedure. Although no formal definition of what constitutes an emergency procedure, guidance states emergency patients are those where indication for surgery is suspected due to either ileus, perforation, bleeding, or otherwise and usually occurs within 36 h of admission. This information was not directly available in the Yorkshire data, so we used surgery within 2 days following an emergency admission. We believe this to be an acceptable way to define an emergency resection, as we compared the estimated proportion in England using our definition, with results from the National Bowel Cancer Audit (NBOCA) over the same time period^36^. The NBOCA used the National Confidential Enquiry into Patient Outcomes and Death (NCEPOD) to classify resections in urgent and emergency procedures^37^. For April 2014 to March 2016, the proportion of English resections we estimated to be an emergency resection was 16.0 per cent, and the proportion of resections in NBOCA classified as urgent and emergency resections was also estimated to be 16.0 per cent. Although the definitions of emergency surgery differ between Denmark and Yorkshire, they are defined in a way that are robust to changes over time, and therefore the observed trend observed in this study should remain valid within both populations.

This study has shown both Denmark and Yorkshire have substantially reduced mortality after surgery in both elective and emergency resections. The larger decrease in overall postoperative mortality in Denmark has coincided with a reduction in the use of emergency resection and increase in stenting procedures, following a policy of converting potential emergency resections into elective resections implemented in some departments. Identifying and addressing the reasons for an increase in emergency surgery in Yorkshire could contribute to a further lowering of postoperative mortality.

## Collaborators

**YCR BCIP Study Group**

The YCR BCIP Study Group includes P.Q., E.J.A.M., P.J.F., N. West (University of Leeds, Leeds, UK), K. Absolom (University of Leeds, Leeds, UK), D. Swinson (Leeds Teaching Hospitals NHS Trust, Leeds, UK), D. Tolan (Leeds Teaching Hospitals NHS Trust, Leeds, UK), S. Howell (University of Leeds, Leeds, UK), S. Alderson (University of Leeds, Leeds, UK), J.C.T., A. Glover (University of Leeds, Leeds, UK), A. Hindley (Leeds Teaching Hospitals NHS Trust, Leeds, UK), H. Rossington (University of Leeds, Leeds, UK), J. Mara (University of Leeds, Leeds, UK) and E. Boldison (University of Leeds, Leeds, UK).

## Supplementary Material

zrac126_Supplementary_Data

## Data Availability

The data used for this study are available via application from the National Cancer Registration and Analysis Service or from UK Colorectal Cancer Intelligence Hub’s CORECT-R, and application to the DCCG, subject to relevant approvals.
