# Supplementary material for: Differences in the management of patients requiring an emergency resection for colonic cancer in two European populations
Source: BJS Open. 2022 Oct 19;6(5):zrac126. doi: 10.1093/bjsopen/zrac126 (PMC9581208; doi:10.1093/bjsopen/zrac126)
Supplement: zrac126_Supplementary_Data [file zrac126_supplementary_data.docx]

**Differences in the management of patients requiring an emergency resection for colon cancer in two European populations**

John C. Taylor^1,3*^, Lene H. Iversen^2^, Dermot Burke^1^, Paul J. Finan^1,3^, Mark M. Iles^1,3^, Eva J.A. Morris^4^, Philip Quirke^1^ on behalf of the YCR BCIP Study Group

^1^ Leeds Institute of Medical Research at St James’s, University of Leeds, Leeds, UK

^2^ Department of Surgery, Aarhus University Hospital, and Danish Colorectal Cancer Group, Aarhus, Denmark

^3^ Leeds Institute for Data Analytics, University of Leeds, Leeds, UK

^4^ Big Data Institute, Nuffield Department of Population Health, University of Oxford, Oxford, UK

**Corresponding author.** J. C. Taylor, Leeds Institute for Data Analytics, Worsley Building, University of Leeds, LS2 9NL, Leeds, UK. Email: [j.c.taylor@leeds.ac.uk](mailto:j.c.taylor@leeds.ac.uk)

**Supplementary Materials - Index**

| **Supplementary Figures and Tables** |  |
| --- | --- |
| Table S1 | *pag. 2* |
|  |  |

**Supplementary Figures and Tables**

| DCCG code | DCCG Operation | Major resection |
| --- | --- | --- |
| 1 | Ileocecal resection | Yes |
| 2 | Right hemicolectomy | Yes |
| 3 | Extended right hemicolectomy | Yes |
| 4 | Resection of traverse colon | Yes |
| 5 | Left hemicolectomy | Yes |
| 6 | Resection of sigmoid colon | Yes |
| 7 | Resection of sigmoid colon with colostomy | Yes |
| 8 | Other combined resection of small intestine and colon | Yes |
| 9 | Other colonic resection without colostomy | Yes |
| 10 | Other colectomy with colostomy and distal closure | Yes |
| 11 | Colectomy and ileorectostomy (anastomosis) | Yes |
| 12 | Colectomy and ileostomy | Yes |
| 13 | Rectal resection (TME and PME) | Yes |
| 14 | Rectal resection + colostomy (=Hartmann’s) | Yes |
| 15 | APE, ischioanal | Yes |
| 16 | APE, extralevator (ELAPE) | Yes |
| 17 | APE, conventional | Yes |
| 18 | APE, intersphincteric | Yes |
| 19 | Proctocolectomy and ileostomy | Yes |
| 20 | Alleviating only (bypass, stoma) | No |
| 21 | Exploration only | No |
| 22 | Transanal endoscopic microsurgery (TEM) | No |
| 23 | Other local procedures incl. polypectomy-EMR | No |
| 24 | Stent in rectum | No |
| 25 | Stent in colon | No |

**Table S1. Operations used to define major resection from the Danish Colorectal Cancer Group (DCCG) database procedure field.**
